# Effect of Exogenous Organic Matter on Phosphorus Forms in Middle-High Fertility Cinnamon Soil

**DOI:** 10.3390/plants13101313

**Published:** 2024-05-10

**Authors:** Xiaodi Shi, Duanyin Gu, Haotian Yang, Yun Li, Yaqun Jiang, Nanbiao Zhan, Xiumin Cui

**Affiliations:** 1 National Engineering Laboratory for Efficient Utilization of Soil and Fertilizer Resources, College of Sources and Environment, Shandong Agricultural University, Tai’an 271018, China; dixiaoshi@126.com (X.S.); jingtianweidi@126.com (Y.L.); dulang@126.com (Y.J.); 15215744145@163.com (N.Z.); 2 Taian Academy of Agricultural Science, Tai’an 271000, China; guduanyin@163.com; 3 College of Agriculture, Shandong Agricultural University, Tai’an 271000, China; zwfy1@sdau.edu.cn

**Keywords:** wheat-maize rotation, available phosphorus, biochar, fulvic acid, soil enzymes, vermicompost

## Abstract

Objectives: To slow down the chemical fixation of phosphate fertilizer, reduce the risk of active phosphorus leaching, stimulate the inherent phosphorus resource activity of soil, and improve phosphorus supply capacity. Methods: This study utilized a combination of field experiments and indoor chemical analysis. Six types of exogenous organic matter (fulvic acid, biochar, compound microbial fertilizer, high-energy microbial inoculum, pig manure-vermicompost, cow manure-vermicompost) were added based on conventional fertilization. The experiment was conducted under the wheat-maize rotation system in the Huang-Huai-Hai region. Results: Compared with control (CK) without exogenous organic matter (EOM), all the other treatments with EOM had an enhancing effect on the available phosphorus of the cultivated soil. During the maize harvest, the combined application of biochar, pig manure-vermicompost and cow manure-vermicompost treatment significantly increased the content of available phosphorus in 0–20 cm soil by 45.87–56.59% compared with CK. The combined application of fulvic acid, biochar, pig manure-vermicompost and cow manure-vermicompost treatment significantly increased the content of Ca_2_-P in 0–20 cm soil by 34.04–65.14%. The content of Ca_10_-P in each treatment with EOM exhibited a lower level compared to CK. EOM could slow down the fixation of phosphorus to some degree. Correlation analysis revealed significant associations between Ca_2_-P, Ca_8_-P, Al-P, Fe-P, neutral phosphatase activity, acid phosphatase activity, and the available phosphorus content in the soil. The combined application of fulvic acid, biochar, and cow manure-vermicompost could enhance the activity of neutral and acid phosphatase in topsoil to a certain extent, thereby facilitating the conversion of phosphorus into highly available Ca_2_-P. EOM could enhance the soil phosphorus availability and decelerate the conversion of soil phosphorus into O-P and Ca_10_-P forms with low availability. Among all treatments, biochar exhibited the most pronounced efficiency in mitigating phosphorus leaching downward. Conclusions: All the EOMs had the potential to enhance the conversion of phosphorus into soluble phosphorus (Ca_2_-P), thereby mitigating the chemical fixation of soil phosphorus and ameliorating non-point source pollution caused by phosphorus. EOM enhanced the activity of neutral and acid phosphatase, which was beneficial to the conversion of organic phosphorus to inorganic phosphorus and increasing the content of available phosphorus. All EOMs had good effects on the retention of soil effective phosphorus, among which biochar had the best effect on retaining effective phosphorus in the tillage layer and blocking phosphorus leaching downward.

Phosphorus (P), an essential nutrient for higher plants, plays an irreplaceable role in crop growth and development as well as in high-yield and high-quality cultivation [1]. The form of phosphorus in the soil includes two categories, inorganic and organic, in which the proportion of inorganic phosphorus is relatively large, accounting for about 60% to 80%, and is the main form of soil phosphorus [2]. Inorganic phosphorus in the soil is the main source of phosphorus uptake by crops, but what could be absorbed and utilized by plants is generally free phosphate ions at very low level. Most phosphorus combines with mineral elements such as calcium, iron, and aluminum in the soil to form a mineral-bound state that is difficult for plants to absorb and utilize [3]. In addition, some of the phosphorus is converted to organic phosphorus, which is considered a potential source of phosphorus and can only be absorbed and utilized by crops after mineralizing to inorganic phosphorus [4]. According to the soil phosphorus grading method, they are classified as calcium phosphate (Ca-P), iron phosphate (Fe-P), aluminum phosphate (Al-P), and closed storage phosphorus(O-P) [5], where calcium phosphate is further classified as dicalcium phosphate (Ca_2_-P), octacalcium phosphate (Ca_8_-P), and decacalcium phosphate (Ca_10_-P) [6]. Phosphorus fertilizer is one of the three key elements of fertilizer, quickly adsorbed and fixed after applying into the soil, converted to slow-acting and high-stability states, which is the main reason for the low utilization efficiency of phosphorus fertilizer in the current season (10% to 25%) [7,8]. Application of phosphorus fertilizer is an important way to increase crop yields, but at present there is a widespread phenomenon of blind application of phosphorus, excessive application in agricultural production, long-term, excessive, high-frequency application, not only resulting in a huge waste of phosphorus fertilizer resources, but also leading to the risk of elevated surface pollution [9]. Under the wheat-maize rotation system of the Huang-Huai-Hai region, which has been cultivated and fertilized for a long time, the soil has formed a huge potential phosphorus storage, and phosphorus enters the ecosystem through runoff and seepage, as a consequence raising the risk of environmental pollution [10]. Research showed that anions in fulvic acid could compete with phosphate for adsorption sites, reducing the adsorption of phosphorus by the soil as well as the immobilization of phosphorus in the soil [11]. Fulvic acid itself has small molecular weight, large activity, and is rich in carboxyl groups, phenolic hydroxyl groups, and other active functional groups, which, on the one hand, can activate the immobilized phosphorus, and on the other hand, can compete for the adsorption site on the soil surface with phosphorus through adsorption or chelating, reducing the immobilization of phosphorus by minerals [12]. Biochar has a rich microporous structure, large specific surface area, and strong adsorption capacity, which can complex with metal ions and promote the dissolution of insoluble phosphorus-containing compounds [8]. Biochar can influence the soil phosphorus by modifying the soil pH, ameliorating the phosphorus complexing metals such as Al^3+^, Fe^3+^ or Ca^2+^, providing a direct source of soluble phosphorus salt, and by promoting the microbial activity and phosphorus mineralization [13]. Microbial fertilizer had a significant effect on available phosphorus by improving the microbial living environment and promoting the transformation of soil nutrients [14]. Microbial inoculum could improve the soil microbial structure around plant roots, increase soil enzyme activity, and increase soil available nutrient content [15]. Vermicompost could significantly increase the number and proportion of soil phosphorus-solubilizing or phosphorus-storing microorganisms [16], thereby increasing soil available phosphorus content. Previous field studies have focused on improving fertilizer efficiency and crop yield, but there are few reports on the effectiveness of EOM on soil phosphorus. In view of the above points, aiming at the rotation mode of wheat-maize in cinnamon soil, this paper studied the changes of phosphorus forms in soil under EOM including fulvic acid, biochar, compound microbial fertilizer, high-energy microbial inoculum, pig manure-vermicompost, and cow manure-vermicompost based on conventional fertilization. The purpose of this paper is to provide the theoretical basis for improving phosphorus utilization rate, activating soil phosphorus storage, and preventing phosphorus non-point source pollution.

## 1. Results and Analysis

### 1.1. Effects of EOM on Soil Phosphatase Activity

#### 1.1.1. Effects of EOM on Soil Neutral Phosphatase Activity

**Figure 1:**
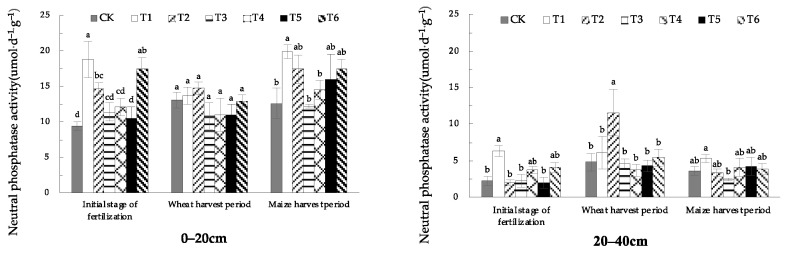
The activity of soil neutral phosphatase with different EOMs. The absence of identical letters within each treatment group across the graph signifies statistically significant differences between the groups (*p* < 0.05).

According to Figure 1, the activity of neutral phosphatase in 0–20 cm soil treated with fulvic acid (T1) was the highest in the initial stage of fertilization and maize-harvest period, which was significantly higher than that in CK by 99.4% and 48.2%, respectively. In the wheat-harvest period, the soil neutral phosphatase activity of the treatment with biochar (T2) was the highest, but there was no significant difference between the treatments. The activity of soil neutral phosphatase was the highest in the treatment of 20–40 cm soil with fulvic acid (T1) in the initial stage of fertilization and maize-harvest period. In the wheat-harvest period, the soil neutral phosphatase activity of biochar (T2) treatment was significantly higher than that in CK, with an increase of 140.8%.

The observed diminution in soil neutral phosphatase activity as a function of increased soil depth was a consistent trend across all experimental treatments, indicating that the phosphatase level in the 20–40 cm soil layer is lower than the 0–20 cm soil layer.

#### 1.1.2. Effects of EOM on Soil Alkaline Phosphatase Activity

**Figure 2:**
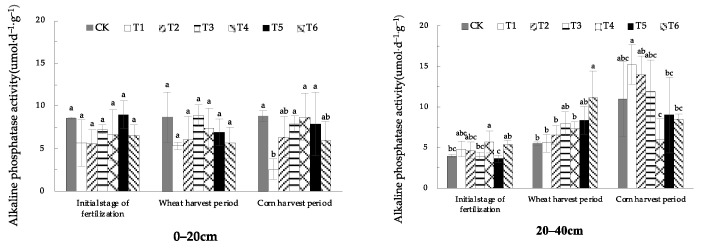
Spraying FA promoted fruit yield of tomato under different Cu, Cd stress. The absence of identical letters within each treatment group across the graph signifies statistically significant differences between the groups (*p* < 0.05).

Seen from Figure 2, there was no significant difference in soil alkaline phosphatase activity between treatments in the initial stage of fertilization and the wheat-harvest period in 0–20 cm soil. In the maize-harvest period, the soil alkaline phosphatase activity with fulvic acid (T1) was the lowest, significantly reduced by 70.9% compared with CK.

From the different soil levels, the alkaline phosphatase activity in the 20–40 cm soil was lower than that in the surface soil in the initial stage of fertilization and showed an increasing trend after the wheat-harvest period and the maize-harvest period, and was generally higher than that in the surface soil after the maize-harvest period, and the difference between treatments became larger.

#### 1.1.3. Effects of EOM on Soil Acid Phosphatase Activity

**Figure 3:**
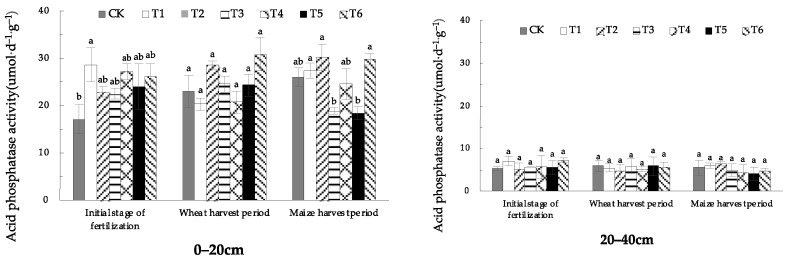
The activity of soil acid phosphatase with different EOMs. The absence of identical letters within each treatment group across the graph signifies statistically significant differences between the groups (*p* < 0.05).

Seen from Figure 3, in the initial stage of fertilization, the soil acid phosphatase activity of 0–20 cm soil treated with fulvic acid (T1) was significantly higher than that in CK. There was no significant difference between each treatment and CK in the wheat-harvest period and the maize-harvest period. The acid phosphatase activity of 20–40 cm soil did not change much and there was no significant difference among the treatments in the initial stage of fertilization, wheat-harvest period and maize-harvest period.

From the different soil layers, the soil acid phosphatase activity in the 20–40 cm soil layer was significantly lower than that in the surface soil and decreased significantly with the increase of soil depth.

### 1.2. Effects of EOM on Soil Available P Content

**Figure 4:**
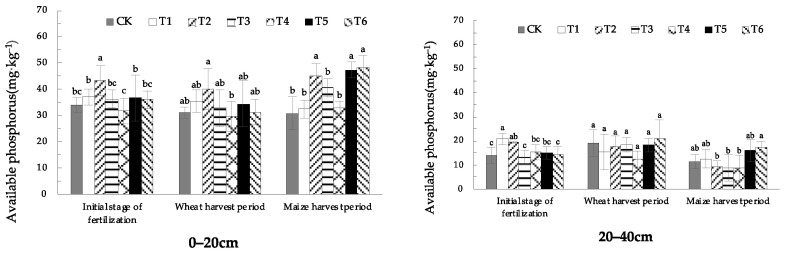
The content of soil available P with different EOMs. The absence of identical letters within each treatment group across the graph signifies statistically significant differences between the groups (*p* < 0.05).

Figure 4 shows that in the 0–20 cm soil, the treatment of biochar (T2) was significantly higher than that in CK in the initial stage of fertilization, wheat-harvest period and maize-harvest period, showing better effective P fixation effect. The treatment of adding pig manure-vermicompost (T5) and cow manure-vermicompost (T6) significantly increased by 34.9% and 39.7%, respectively, compared with CK in the maize-harvest period. In general, the content of available P in the soil increased rapidly in the initial stage of fertilization. Compared with CK, EOM could activate the soil inherent P storage in short term and increase the available P content in the soil. During the wheat-harvest period, after eight months of transformation and the growth and absorption of a crop of wheat, the content of soil available P in each treatment decreased. There was no significant effect difference between the 0–20 cm and 20–40 cm soil layers after the treatment of high-energy microbial inoculum (T4) was applied to the soil, and the available P content in the soil was low.

From the different soil layers, with the deepening of the soil layer, the soil available P took a decreasing trend. The higher the content of available P in the surface layer, the higher was the content of available P in the deep layer of the treatment, indicating that there was a certain degree of leaching of available P. The soil treated with biochar (T2) had a high content of available P in the surface soil, but the content of available P in the deep soil was low, indicating that biochar could effectively block leaching of phosphorus.

### 1.3. Effects of EOM on the Content of Inorganic P in Soil

#### 1.3.1. Effects of EOM on Soil Ca_2_-P Content

**Figure 5:**
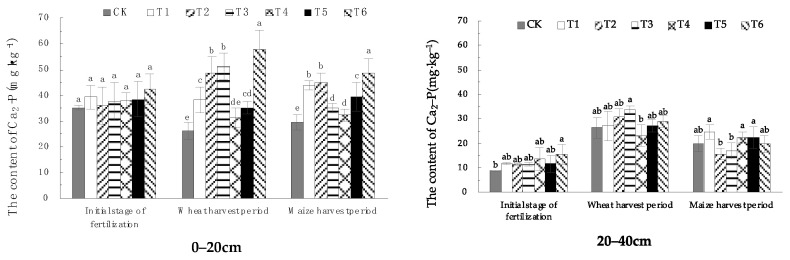
The content of soil Ca_2_-P with different EOMs. The absence of identical letters within each treatment group across the graph signifies statistically significant differences between the groups (*p* < 0.05).

Seen from Figure 5, the Ca_2_-P content of the 0–20 cm soil in the initial stage of fertilization was around 35 mg·kg^−1^, and there was no significant difference between the treatments. In the wheat-harvest period, except for the treatment with high-energy microbial inoculum (T4), the Ca_2_-P content of other EOMs was significantly higher than that in CK, and the Ca_2_-P content of soil treated with cow manure-vermicompost (T6) was the highest, with a 135.6% increase compared with CK, and the difference was highly significant. In the maize-harvest period, soil Ca_2_-P content of all treatments of EOM was significantly higher than that in CK, and the soil Ca_2_-P content of cow manure-vermicompost(T6) treatment was the highest, with an increase of 68.0%.

From the distribution trend of Ca_2_-P in different soil layers, the downward shift of leaching was significantly enhanced during the wheat-harvest period and the maize-harvest period compared with the initial stage of fertilization. The soil Ca_2_-P content of treatments with EOM was generally higher than that in CK in both soil horizons of 0–20 cm and 20–40 cm, which indicated the EOM promoted the conversion of phosphorus into Ca_2_-P to a certain extent.

#### 1.3.2. Effect of EOM on Ca_8_-P Content

The soil Ca_8_-P content of the 0–20 cm surface soil was significantly lower than that in CK only in the treatment with fulvic acid (T1) in the wheat-harvest period. In the maize harvest period, the soil Ca_8_-P content was highest in the treatment with pig manure-vermicompost (T5), increasing by 23.1% compared with CK. The Ca_8_-P content of the soil in the 20–40 cm soil did not change much in the initial stage of fertilization, the wheat-harvest period and the maize-harvest period. Only the treatment with fulvic acid (T1) was lower than the other treatments in the wheat-harvest period. The other treatments showed a decreasing trend throughout the growing season.

**Figure 6:**
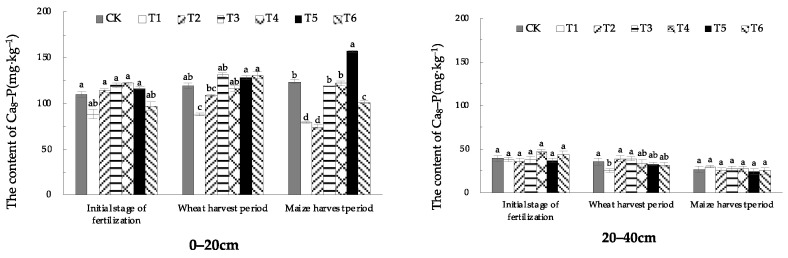
The content of soil Ca_8_-P with different EOMs. The absence of identical letters within each treatment group across the graph signifies statistically significant differences between the groups (*p* < 0.05).

From different soil layers, Ca_8_-P in the 20–40 cm soil layer was significantly lower than that in the 0–20 cm surface layer, and the degree of leaching downward was lower, and the content only ranged from 19.2% to 44.8% of that in the surface soil layer (Figure 6).

#### 1.3.3. Effect of EOM on Soil Al-P Content

**Figure 7:**
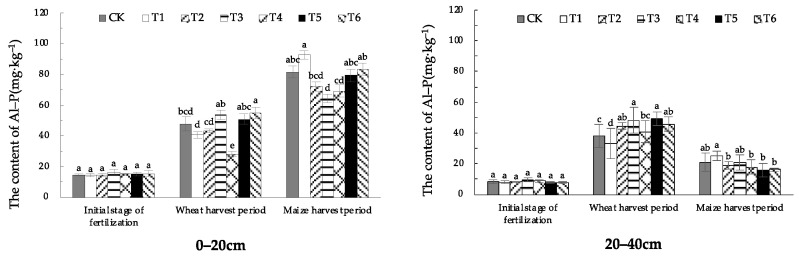
The content of soil Al-P with different EOMs. The absence of identical letters within each treatment group across the graph signifies statistically significant differences between the groups (*p* < 0.05).

The Al-P content of the treatments in 0–20 cm soil was the lowest in the initial stage of fertilization, increased by 84.9–259.6% in the wheat-harvest period. The overall further increased in the maize-harvest period, with obvious accumulation from the initial stage of fertilization, increased 28.5% to 100.1%. In the growing period, in the wheat-harvest period, the treatment with cow manure-vermicompost (T6) had the highest soil Al-P content, significantly increased by 15.4% compared with that of CK. The soil treated with high-energy microbial inoculum (T4) had the lowest Al-P content, which was significantly lower than that of CK. Up to the maize-harvest period, the Al-P content of the 0–20 cm soil in the compound microbial fertilizer treatment (T3) was significantly lower than that of CK. Conversely, the analysis revealed an absence of significant disparity in the Al-P content of the two soil layers in treatments with other EOMs compared to CK (Figure 7). From the whole fertility stage of the wheat and maize, the Al-P content of all treatments in 20–40 cm soil was the highest in the wheat-harvest period, increased by 314.6–524.5% compared with the initial stage of fertilization, and by 32.5–209.7% compared with the maize-harvest period. Among which, the pig manure-vermicompost (T5) was the most obvious enhancement, 524.5% and 209.7% higher than that in the initial stage of fertilization and the maize-harvest period. In 0–20 cm soil, biochar (T2) was lower than that in CK in all stages, while cow manure-vermicompost (T6) was higher than that in CK in all stages. In 20–40 cm soil, only the treatment with fulvic acid (T1) had lower soil Al-P content than that in CK in the wheat-harvest period, and all the other treatments were higher than that in CK, while only the treatment with fulvic acid (T1) had higher soil Al-P content than that in CK in the maize-harvest period, and all the other treatments were lower than that in CK. In the harvest period of maize, only in the treatment T1, the soil Al-P content was higher than that in CK.

From different soil layers, the Al-P content in the 20–40 cm soil layer was significantly lower than that in the 0–20 cm layer during the maize-harvest period, with a lower degree of leaching downward.

#### 1.3.4. Effect of EOM on Soil Fe-P Content

The soil Fe-P content of 0–20 cm soil in the treatment with fulvic acid (T1) was significantly higher than that in CK in the initial stage of fertilization. In the wheat-harvest period, the soil Fe-P content of EOM treatments was significantly higher than that in CK, except for the treatment with composite microbial fertilizer (T3). The soil Fe-P content of the treatment with added cow manure-vermicompost (T6) was the highest, increased by 49.9% compared with CK. In the maize-harvest period, the soil Fe-P content of fulvic acid (T1), biochar (T2), and cow manure-vermicompost (T6) treatments were increased by 23.7%, 18.5%, and 12.5%, respectively, compared with CK. The soil from 20–40 cm was higher than that in CK in the initial stage of fertilization except for the treatment of pig manure-vermicompost (T5). The Fe-P content showed an increment of 15.8% compared to CK. In the stage of wheat harvest, Fe-P content of all treatments with EOM were lower than that in CK, except for the amendment of biochar (T2) and cow manure-vermicompost (T6) treatments that reached CK level, in which the soil Fe-P content of the soil treated with fulvic acid (T1), composite microbial fertilizer (T3), high-energy microbial inoculum (T4), and pig manure-vermicompost (T5) was reduced by 21.7%, 23.4%, 29.0%, and 15.3%, respectively, compared with CK. In the maize-harvest period, the soil Fe-P content of all EOM treatments was higher than that in CK, except for the treatment with compound microbial fertilizer (T3), and the soil Fe-P content of the treatment with fulvic acid (T1) was the highest, increased by 29.0% compared with CK.

**Figure 8:**
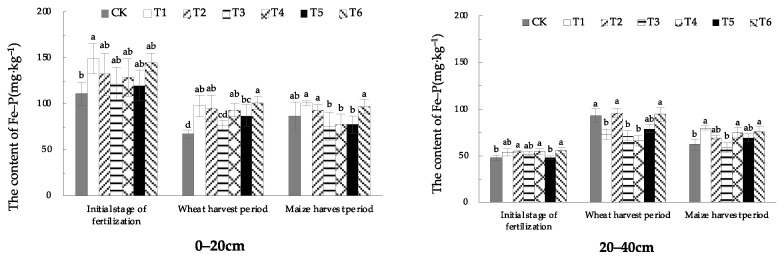
The content of soil Fe-P with different EOMs. The absence of identical letters within each treatment group across the graph signifies statistically significant differences between the groups (*p* < 0.05).

The distribution trend of Fe-P in different soil layers showed that the downward shift of leaching was significantly enhanced during the wheat-harvest period and the maize-harvest period compared to the initial stage of fertilization (Figure 8).

#### 1.3.5. Effect of Different EOMs on Soil O-P Content

**Figure 9:**
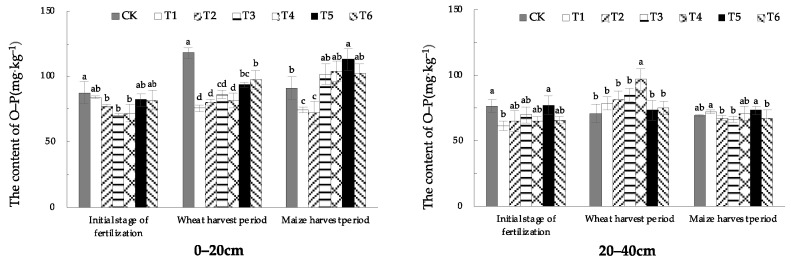
The content of soil O-P with different EOMs. The absence of identical letters within each treatment group across the graph signifies statistically significant differences between the groups (*p* < 0.05).

In the initial stage of fertilization in 0–20 cm soil, the soil O-P content treated with EOM was lower than that in CK. In the wheat-harvest period, the soil O-P content of treatments with EOM was significantly lower than that in CK, among which the O-P content treated with fulvic acid (T1) was the lowest, decreased by 35.9% compared with CK. In the maize-harvest period, soil O-P content treated with fulvic acid (T1) and biochar (T2) was significantly lower than in CK. The soil O-P content of 20–40 cm soil was significantly lower than that in CK in the initial stage of fertilization, except for pig manure-vermicompost (T5), the soil O-P content of all treatments with EOM had different decreases. In the wheat-harvest period, soil O-P content of all treatments with EOM had an increasing tendency, only the treatment of high-energy microbial inoculum (T4) was significantly higher. In the maize-harvest period, there was no significant difference in O-P content between EOM treatments and CK (Figure 9).

From the overall trend in different soil layers, the soil O-P contents in the 0–20 cm and 20–40 cm layers were relatively stable. However, in the initial stage of fertilization, the soil O-P content of the treatments with EOM was generally lower than that in CK, which showed that EOM could slow down the conversion of phosphorus to closed storage phosphorus O-P.

#### 1.3.6. Effect of EOM on Soil Ca_10_-P Content

In 0–20 cm soil, in the initial stage of fertilization and wheat-harvest period, the soil Ca_10_-P content with EOM was not significantly different from CK. In the maize-harvest period, the soil Ca_10_-P content of EOM treatments became lower, and soil Ca_10_-P content of the fulvic acid (T1) treatment was the lowest, significantly reduced by 19.0%. In the 20–40 cm soil layer, in the initial stage of fertilization, the soil Ca_10_-P content of the fulvic acid (T1) treatment was significantly lower than that in CK. In the wheat-harvest period, the soil Ca_10_-P content of the high-energy microbial inoculum (T4) treatment was significantly lower. In the maize-harvest period, there was no statistically significant difference between the treatments and CK.

**Figure 10:**
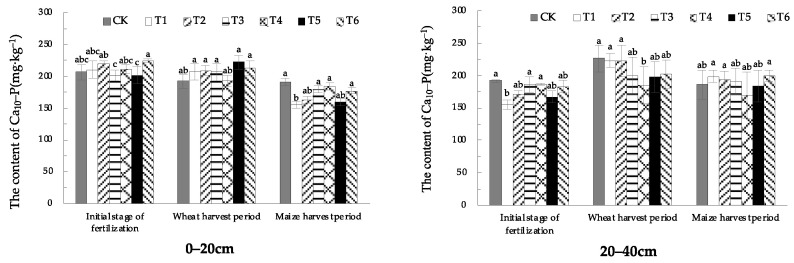
The content of soil Ca_10_-P with different EOMs. The absence of identical letters within each treatment group across the graph signifies statistically significant differences between the groups (*p* < 0.05).

From the different soil layers, soil Ca_10_-P content was around 200 mg·kg^−1^ in both horizons, with a high level and overall stable state (Figure 10).

### 1.4. Correlation Analysis of Different Phosphorus Forms with Phosphatase Activity

Correlation analysis showed (Table 1) that soil effective phosphorus correlated with soil Ca_2_-P, Ca_8_-P, Al-P, Fe-P, neutral phosphatase, and acid phosphatase to highly significant levels, and did not correlate significantly with O-P, Ca_10_-P, and alkaline phosphatase. Ca_2_-P reached highly significant correlations with Ca_8_-P, Al-P, Fe-P, O-P, neutral phosphatase, and acid phosphatase, and no significant correlation with Ca_10_-P and alkaline phosphatase. Ca_8_-P reached highly significant correlations with O-P, Fe-P, neutral phosphatase, acid phosphatase, and no significant correlations with Al-P, Ca_10_-P, alkaline phosphatase. Al-P reached highly significant correlations with Fe-P, Ca_10_-P, acid phosphatase, a significant correlation with neutral phosphatase, and no significant correlation with O-P and alkaline phosphatase. Fe-P reached highly significant correlations with Ca_10_-P, neutral phosphatase, and acid phosphatase, and no significant correlations with O-P and alkaline phosphatase. O-P reached a highly significant correlation with Ca_10_-P, neutral phosphatase, and no significant correlation with acid phosphatase and alkaline phosphatase. Ca_10_-P had no significant correlation with phosphatase. Neutral phosphatase had a highly significant correlation with acid phosphatase and no significant correlation with alkaline phosphatase. Alkaline phosphatase had no significant correlation with acid phosphatase. This indicated that increasing the soil Ca_2_-P, Ca_8_-P, Al-P and Fe-P content could effectively improve the effective phosphorus content. Acidic and neutral phosphatases were closely related to soil phosphorus availability.

## 2. Materials and Methods

### 2.1. General Description of the Experiment Site

The experiment was carried out in Shiqiang Town, Zoucheng City, Jining City, Shandong Province, China (E 116°89′–116°90′, N 35°29′–35°30′), from October 2018 to October 2019. This field had a warm temperate monsoon climate with an average annual precipitation of 684.8 mm. The main planting pattern was winter wheat/summer maize one-year double cropping system. The area’s soil is classified as Cambisols (according to the World Soil Classification System, WRB) and Cinnamon soils (according to the Chinese Soil Classification System) [17]. The physical and chemical properties of the tested soil are displayed in Table 2. According to the nutrient classification standard of China’s second soil census (the environmental risk threshold of phosphorus is 40 mg·kg^−1^) [18], the content of available phosphorus in 0–20 cm soil layer was high, and the content of available phosphorus in 20–40 cm soil layer was medium.

### 2.2. Test Materials

The wheat variety was ‘Tainong 18’. The maize variety was ‘Zhengdan 958’. The fertilizers used in the experiments were from Shandong Agricultural University Fertilizer Sci.&tech. Co., Ltd., Feicheng City, China. compound fertilizer (N-P_2_O_5_-K_2_O 19-18-18), urea (N 46%), diammonium phosphate (N 18%, P_2_O_5_ 46%), potassium chloride (K_2_O 51%). EOM used in the tests: Fulvic acid (Shandong Quanlin Jiayou Co., Ltd., Liaocheng, China), Biochar (Liaoning Province Biochar Engineering Technology Research Center, Shenyang, China), compound microbial fertilizer (Jiangyin Lianye Biotechnology Co., Ltd., Jiangyin, China), high-energy microbial inoculum (Yunnan Xixing Agricultural Technology Co., Ltd., Kunming, China), pig manure-vermicompost and cow manure-vermicompost (Jiangxi Guiyi Agricultural Technology Co., Ltd., Shangrao, China). The experimental design is shown in Table 3.

The appropriate amount of organic matter was determined based on the results of the previous trials and relevant literature [19,20], with the same amount used in the wheat and maize seasons. The plot area was 5 m × 10 m = 50 m^2^, with a 2-m isolation belt in the middle, three repetitions, randomly arranged.

The general fertilization amount of wheat was N 225 kg·hm^−2^, P_2_O_5_ 120 kg·hm^−2^, K_2_O 105 kg·hm^−2^. The required potassium was applied in the form of compound fertilizer as a one-time basal fertilizer. The remaining phosphorus accounted for 12.5% of the total phosphorus, and the remaining nitrogen accounted for 50% of the total nitrogen. Diammonium phosphate and urea were used as topdressing supplements, respectively. After maize harvest, all straws were returned to the field (about 11,000 kg·hm^−2^). The basal fertilizer was thoroughly mixed with the corresponding EOM (fulvic acid, biochar, compound microbial fertilizer, high-energy microbial inoculum, pig manure-vermicompost and cow manure-vermicompost). Subsequently, this integrative mixture is disseminated evenly and incorporated into the soil through deep tilling, reaching an approximate depth of 30 cm. The seeding quantity of the wheat was 165 kg·hm^−2^, sowed around October 5th, harvested around June 5th the next year, wide precision sowed, row spacing was 27 cm, growth period was about 230 days.

The fertilizers used in the maize season were urea, diammonium phosphate and potassium sulfate, equivalent to N 255 kg·hm^−2^, P_2_O_5_ 45 kg·hm^−2^, and K_2_O 60 kg·hm^−2^. After the wheat harvest, the soil was not tilled, and the corresponding EOM was mixed with the fertilizer and then applied all at once, and the seeds were applied. The planting density of the maize was 72,000 plants·hm^−2^. Sowed around June 20th, harvested around October 3rd, the growth period was about 100 days. The management of the experimental plot was the same as that of a local conventional field.

### 2.3. Sample Collection and Determination

The soil at 0–20 cm and 20–40 cm depth was collected in the initial stage of fertilization (28 October 2018), the wheat-harvest period (31 May 2019), and the maize-harvest period (3 October 2019). Soil samples were taken between the wheat rows and between the maize plants, using the “W” sampling method, with a soil drill to collect 10 points in each plot. The soil was fully mixed, and about 300 g of the soil was taken back to the laboratory in a plastic bag, then naturally air-dried, grinded, sifted, preserved, and kept in reserve.

Soil test indicators: pH, available phosphorus content, total inorganic phosphorus, Ca_2_-P, Ca_8_-P, Al-P, Fe-P, O-P, Ca_10_-P [21], neutral phosphatase activity, acid phosphatase activity, alkaline phosphatase activity.

Soil specimens were blended with distilled water at a ratio of 1:2.5 (*w/v*, soil to water), followed by oscillating for 1 min and static setting for 30 min. The soil pH was assessed using a pH meter (PHS-3C laboratory pH meter, Shanghai).

The available phosphorus was extracted with 0.5 mol·L^−1^ NaHCO_3_ at pH 8.5. Total inorganic phosphorus was extracted by the SMT method, 0.2 g soil was taken in a 50 mL centrifuge tube, and added with 20 mL of 1 mol·L^−1^ HCl, oscillated for 30 min, centrifuged at 3000 r·min^−1^ for 10 min. The content of the various forms of inorganic phosphorus in soil was determined by the grading method. Ca_2_-P (NaHCO_3_ soluble phosphorus) was extracted by 0.25 mol·L^−1^ NaHCO_3_. Ca_8_-P (NH_4_OAc soluble phosphorus) was extracted with 0.5 mol·L^−1^ NH_4_OAc. Al-P (NH_4_F soluble phosphorus) was extracted with 0.5 mol·L^−1^ NH_4_F. Fe-P (NaOH-Na_2_CO_3_ soluble phosphorus) was extracted by 0.1 mol·L^−1^ NaOH-0.1 mol·L^−1^ Na_2_CO_3_. O-P (occluded phosphorus) was extracted with 0.3 mol·L^−1^ sodium citrate-0.5 mol·L^−1^ sodium hydroxide solution and determined after digestion. Ca_10_-P (H_2_SO_4_ soluble phosphorus) was extracted with 0.5 mol·L^−1^ H_2_SO_4_. Two drops of dinitrophenol indicator were added to each extracted P fraction solution, and the pH was adjusted with HCl and NaOH until the solution just turned yellow. Ammonium molybdate [(NH_4_)_6_Mo_7_O_24_·4H_2_O] solution containing ascorbic acid (5 mL) was added to each mixed solution and then diluted to 50 mL. Samples were allowed 30 min for color development. The absorbance of the solutions at 880 nm was measured with the UV–visible spectrophotometer (UV-5500, Shanghai, China).

The activity of soil acid phosphatase, neutral phosphatase, and alkaline phosphatase was determined by micro-determination (kit from Suzhou Keming Biotechnology Co., Ltd., Suzhou, China).

### 2.4. Data Processing

The data were processed and plotted by Word, Excel, and Origin, and were statistically analyzed with SPSS. The Duncan multiple comparison method was used for significance analysis (*p* < 0.05).

## 3. Discussion

### 3.1. Effect of EOM on Phosphorus Speciation

Phosphorus is an essential nutrient element for crop growth, and one of the three fertilizers necessary for maintaining high yield and high efficiency in agriculture [22]. Phosphorus in soil mainly exists in the form of inorganic phosphorus, which is the main source of phosphorus absorption by plants. Organic phosphorus is a potential phosphorus pool, which can also be absorbed and utilized after activation by organic components or microorganisms. Soil available phosphorus refers to the general term of phosphorus that plants can absorb and utilize, which mirrors the soil’s capacity to furnish phosphorus to plants. In recent years, the accumulation of phosphorus in soil caused by excessive fertilizer application has led to nutrient imbalance, phosphorus eluviation, and eutrophication [23]. Therefore, reducing the input of phosphorus from the source, activating the inherent phosphorus resources of the soil, and improving the utilization rate of phosphorus are the necessary ways to achieve high quality and efficiency of the soil and improve the soil environment. Studies have shown that some EOMs can effectively slow down the fixation of phosphorus in soil and enhance its effectiveness. Studies have shown that biochar can potentially increase soil phosphorus availability through direct mechanisms or phosphorus adsorption from fertilizers [24]. The application of biochar has been identified as a significant factor in altering soil phosphorus solubilization, attributed to biochar’s capacity to impede the binding of various ions to soluble phosphorus [25]. Studies have shown that fulvic acid can increase the solubility of phosphorus in soil [26]. High-energy microbial inoculum are the main active beneficial bacteria, which stimulate the growth of the bacterial population, improve the composition of microbial community and soil nutrient status, and increase soil enzyme activity and soil available phosphorus content [27]. Vermicompost is rich in organic matter, which stimulates the growth of soil microorganisms and promotes the transformation of soil phosphorus to the available state [16]. In this research, in terms of soil effective phosphorus content, except for high-energy microbial inoculum treatment, all EOM treatments increased the available phosphorus content in surface soil to varying degrees during the wheat and corn harvests. Biochar had the best effect on the fixation of available phosphorus. In the initial stage of fertilization and during both two harvest periods, the available phosphorus in the surface layer increased significantly. There was little change in the effective phosphorus content of the high-energy microbial inoculum treatment, which may be related to the dosage of matter.

Studies have shown that the application of rice straw biochar can effectively increase the content of Ca_2_-P, Fe-P, and Al-P in soil [8], and the continuous application of biochar in brown soil can increase the content of Ca_2_-P, Ca_8_-P and Al-P in soil [28]. Due to the rich pore structure of biochar, it can adsorb phosphate ions [29] and increase Ca_2_-P content through adsorption and desorption. In addition, biochar itself contains more trace elements, such as Fe and Mg, which can enhance phosphorus adsorption by iron oxides and aluminum oxides, increasing Fe-P content. In this research, the treatment of EOM biochar promoted the increase of Ca_2_-P and Fe-P content in the surface soil during the wheat-harvest period and maize-harvest period, especially in the surface soil during the wheat-harvest period, and the effect was extremely significant. The content of Ca_2_-P increased by 86.3% compared with CK. It has been found that fulvic acid competes with phosphate for adsorption sites on the soil surface through adsorption or chelation, reducing the adsorption and fixation of phosphate by minerals [30]. In this research, EOM fulvic acid effectively increased the content of Ca_2_-P and Fe-P in wheat- and maize-harvest periods, slowed down the conversion of phosphorus to O-P and Ca_10_-P, and improved soil phosphorus availability. Studies have shown that vermicompost with fertilizer significantly affects the increase of the soil’s active phosphorus content, which is conducive to slowing down the transformation of active phosphorus to steady-state phosphorus [16]. In this research, compared with conventional fertilization, the contents of Ca_2_-P, Ca_8_-P and Al-P in plow-layer soil of pig manure-vermicompost treatment increased to varying degrees during the wheat- and maize-harvest periods, and Ca_8_-P increased by 27.5% during the maize-harvest period. The contents of Ca_2_-P, Al-P and Fe-P in plow-layer soil of cow manure-vermicompost treatment increased in both wheat and maize-harvest. The contents of Ca_2_-P, which were easily absorbed by crops, increased significantly by 121.5% and 65.1%, respectively. In the wheat-harvest period, the O-P content in the surface soil of pig manure-vermicompost and cow manure-vermicompost treatments decreased, which may be related to the production of more organic acids after the application of vermicompost into the soil. The organic acids produced by the decomposition of vermicompost provide a reducing environment, which weakens the oxidation rate of free ferrous ions in ferrous phosphate, hinders the pathway of ferrous phosphate and aluminum phosphate to wrap iron oxide, thereby reducing the O-P content and improving the effectiveness of phosphorus. Vermicompost itself contains phosphorus, which enriches the diversity of soil beneficial microorganisms [31], thus promoting the transformation of phosphorus into a form that is easily absorbed by roots [32], resulting in an increase in Ca_2_-P content [33]. The increase of soil O-P content in earthworm manure treatment during maize harvest may be related to the low soil temperature and humidity during this period. In this study, there was no significant change in the content of various forms of phosphorus in the soil treatment with high-energy microbial inoculum and compound microbial fertilizer. This may be related to EOM high-energy biological inoculum or the slow process of increasing phosphorus availability only by changing microbial activity.

It has been suggested that Ca_2_-P, Ca_8_-P, and Al-P were effective phosphorus sources in the inorganic form [34], whereas Ca_2_-P, Ca_8_-P, Al-P, and Fe-P were highly significantly correlated with available phosphorus in this research, and the above four forms of phosphorus were all effective phosphorus sources. Therefore, the increase of soil available phosphorus content in the treatment of EOM fulvic acid, biochar, pig manure-vermicompost, and cow manure-vermicompost was largely due to the increase of soil inorganic phosphorus available phosphorus source, which slowed down the conversion of phosphorus to O-P and Ca_10_-P, showing an excellent activation effect.

### 3.2. Effect of EOM on Soil Phosphatase Activity

The conversion of organic phosphorus in soil is influenced by several factors, especially the involvement of phosphatases, which accelerate the conversion of organic phosphorus to efficient inorganic phosphorus [35]. A variety of phosphatases are present in soils with pH 4–9 and they play an important role in the activation and maintenance of soil phosphorus effectiveness. Soil phosphatase regulates the storage form of soil phosphorus, and its activity level directly affects the decomposition and bioavailability of soil organic phosphorus [36]. In this research, effective phosphorus was highly correlated with neutral and acid phosphatases, but not significantly correlated with alkaline phosphatase. Application of fulvic acid significantly increased the activity of neutral and acid phosphatases in the surface soil in the initial stage of fertilization, while the activity of neutral phosphatases increased significantly during the maize-harvest period. Studies have shown that an appropriate amount of biochar can significantly increase the activity of acid phosphatase [8]. In this research, compared with CK, EOM fulvic acid, biochar, and cow manure-vermicompost treatments all increased surface soil neutral and acid phosphatase activities to some extent. Correlation analysis showed that neutral phosphatase and acid phosphatase were highly significantly correlated with effective phosphorus, Therefore, EOM fulvic acid, biochar, and cow manure-vermicompost had a positive effect on promoting the transformation of soil organic phosphorus to inorganic phosphorus and increasing the content of effective phosphorus, which may be closely related to the improvement of the living environment of soil microorganisms and the enhancement of the vital activities of phosphate solubilizing microorganisms [8,11,30].

### 3.3. Effect of EOM on the Blockage of Phosphorus Leaching

As a key factor of water eutrophication, phosphorus leaching in the wet season is an important factor in the deterioration of the water ecosystem and the decline of soil quality in Huang-Huai-Hai irrigation area [37,38]. Therefore, loss control is the fundamental way to prevent phosphorus non-Point source pollution. In this study, different EOMs had different effects on soil phosphorus forms at different depths. There was some leaching of Ca_2_-P during wheat harvest and maize harvest, and the downward movement of leaching increased significantly with time. Ca_8_-P was less mobile and less downward leached in different soil layers. Compared with the surface layer, the Al-P content was significantly lower in the 20–40 cm soil layer during the maize-harvesting period, indicating that phosphorus leaching was not significant in this form. The Fe-P content in the 20–40 cm of soil was significantly lower than that in the surface layer in the initial stage of fertilization, whereas it was higher in 20–40 cm of soil during the harvesting period of wheat and maize, suggesting that the degree of Fe-P loss was higher during the hot and rainy season. Soil O-P content was relatively stable in the 0–20 cm and 20–40 cm soil layers, and compound fertilizer application converted phosphorus to O-P, a closed storage state of phosphorus, with EOM somewhat blocking this trend. The higher the effective phosphorus content in the surface layer of the soil, the higher the effective phosphorus content in the deeper layers, indicating some degree of effective phosphorus leaching. In the case of high available phosphorus content in the surface layer, the available phosphorus content in the deep layer was lower in the treatment with EOM biochar. Such a situation suggests that the combined application of biochar and base fertilizer could effectively fix the available phosphorus in the rhizosphere of the soil and prevent the loss of available phosphorus in the surface layer of the soil.

## 4. Conclusions

(1) EOM promoted the conversion of phosphorus to moderately soluble phosphorus (Ca_8_-P, Al-P, Fe-P), and slowed down the conversion of phosphorus to closed-accumulation phosphorus O-P and mineralized phosphorus Ca_10_-P to a certain extent. 4000 kg·hm^−2^ of cow manure-vermicompost treatment was the best for the promotion of the conversion of phosphorus to Ca_2_-P in the surface layer of the soil.
(2) EOM played a positive role in increasing the activity of soil neutral and acid phosphatase, promoting the conversion of organic phosphorus to inorganic phosphorus, and increasing the content of effective phosphorus, and 59.97 kg·hm^−2^ fulvic acid treatment was generally more effective.
(3) Fulvic acid, biochar, microbial fertilizer, cow manure-vermicompost and pig manure-vermicompost were all effective in activating and retaining effective phosphorus in the soil. 900 kg·hm^−2^ biochar treatment was the most effective in preventing downward phosphorus loss from the surface layer.

## Figures and Tables

**Table 1:** Correlation analysis between different phosphorus forms and phosphatase. ** Significant correlation at the 0.01 level (two-sided); * Significant correlation at the 0.05 level (two-sided).

Type	Available Phosphorus	Ca_2_-P	Ca_8_-P	Al-P	Fe-P	O-P	Ca_10_-P	Neutral Phosphatase	Alkaline Phosphatase	Acid Phosphatase
Available phosphorus	1									
Ca_2_-P	0.697 **	1								
Ca_8_-P	0.676 **	0.714 **	1							
Al-P	0.667 **	0.627 **	0.288	1						
Fe-P	0.678 **	0.512 **	0.579 **	0.664 **	1					
O-P	0.209	0.458 **	0.415 **	−0.243	−0.110	1				
Ca_10_-P	−0.257	0.018	−0.124	−0.409 **	−0.434 **	0.674 **	1			
Neutral phosphatase	0.717 **	0.820 **	0.845 **	0.346 *	0.474 **	0.584 **	0.016	1		
Alkaline phosphatase	−0.070	−0.067	−0.221	0.103	−0.071	0.071	0.140	−0.213	1	
Acid phosphatase	0.685 **	0.826 **	0.858 **	0.470 **	0.520 **	0.362 *	−0.157	0.922 **	−0.274	1

**Table 2:** Soil nutrient fundamentals.

Soil Depth (cm)	Organic Matter (g·kg^−1^)	Total Nitrogen (g·kg^−1^)	Alkaline Hydrolyzable Nitrogen (mg·kg^−1^)	Available Phosphorus (mg·kg^−1^)	Available Potassium (mg·kg^−1^)	pH
0–20	18.57	0.49	83.84	40.18	154.5	7.10
20–40	10.27	0.30	55.57	18.89	121.3	

**Table 3:** The treatments in this experiment.

Treatment Code	Treatment
CK	conventional fertilization (other treatments were added based on CK)
T1	fulvic acid (59.97 kg·hm^−2^)
T2	biochar (900 kg·hm^−2^)
T3	compound microbial fertilizer (748 kg·hm^−2^)
T4	high-energy microbial inoculum (748 kg·hm^−2^)
T5	pig manure-vermicompost (4000 kg·hm^−2^)
T6	cow manure-vermicompost (4000 kg·hm^−2^)

## Data Availability

Data are contained within the article.
